# Comparison of simultaneous vs sequential pars plana vitrectomy and cataract surgery

**DOI:** 10.1186/s12886-023-02801-y

**Published:** 2023-02-23

**Authors:** Abdelhalim A. Awidi, Priya M. Mathews, Nakul Shekhawat, Fasika A. Woreta, Divya Srikumaran, Yassine J. Daoud

**Affiliations:** Cornea, Cataract and Anterior Segment Division, The Wilmer Eye Institute, Johns Hopkins Hospital, The Johns Hopkins University School of Medicine, 600 N Wolfe Street, Maumenee 327, Baltimore, MD 21287 USA

**Keywords:** Cataract surgery, Pars plana vitrectomy, Simultaneous, Sequential, Outcomes

## Abstract

**Background:**

To compare the clinical outcomes of patients undergoing sequential pars plana vitrectomy (PPV) followed by cataract extraction surgery (CE) [PPV/CE], simultaneous PPV and CE (PPV + CE), and sequential CE followed by PPV [CE/PPV].

**Methods:**

A retrospective observational cohort study of 427 eyes of 404 patients who underwent either sequential or simultaneous PPV and CE surgery between March 2016 and May 2021. Pre-operative and post-operative assessments (up to 2 years of follow-up visits) of uncorrected visual acuity (UCVA), corrected distance visual acuity (CDVA), spherical equivalent (SEQ), and refractive prediction error (RPE) was done. Main outcome measures were both visual (UCVA, CDVA) and refractive (RPE, SEQ).

**Results:**

There was a statistically significant difference in CDVA of the PPV/CE, PPV + CE, CE/PPV groups (logMAR 0.34 ± 0.40, 0.65 ± 0.61, and 0.55 ± 0.60, respectively) at one month postoperatively (POM1) (*P* < 0.001), and at the POM12 visits (logMAR 0.25 ± 0.34, 0.53 ± 0.68, and 0.44 ± 0.48; *P* = 0.04). In the subgroup analysis of patients with a diagnosis of either epiretinal membrane or vitreous opacities, there was no statistically significant difference in SEQ (*P* = 0.09) and RPE (*P* = 0.13) at the combined 1 month and 3 month visits.

**Conclusion:**

Simultaneous PPV and cataract surgery demonstrated similar improvements in visual acuity and refractive outcomes, as well as comparable intraoperative and postoperative complication profiles to sequential surgery.

## Introduction

In a subset of cataract patients, concomitant posterior segment disease warrants the combination of pars plana vitrectomy (PPV) and cataract surgery. Performing simultaneous PPV and cataract removal may be beneficial since hastened cataract progression is one of the most common complications in patients undergoing PPV [1, 2]. Other possible advantages of simultaneous surgery include the ability to avoid accidental lens touch with vitrectomy instruments, and enhanced visualization of the posterior segment for membrane peeling and vitrectomy [3]. Apart from minimizing the added risks of intra and postoperative complications associated with increased trips to the operating room, simultaneous surgery leads to decreased clinic visits and the associated burden on the patient and the family. On the other hand, simultaneous surgery may have drawbacks such as difficulty coordinating the schedules of vitreoretinal and cataract surgeons. Furthermore, a longer procedure time may cause intraoperative corneal edema, increased postoperative inflammation and intraocular pressure, higher rate of synechia, and cystoid macular edema [4–6].

Performing a sequential PPV followed by cataract surgery can also have advantages, particularly in patients with vitreous hemorrhage that may have a decreased red reflex prior to PPV. Limitations to sequential surgery include visual acuity decline due to delayed onset cataract formation following PPV. Higher rates of intraoperative lens-iris diaphragm retropulsion syndrome and posterior capsule rupture in patients undergoing sequential surgery have also been reported [7, 8].

On occasion, a vitreoretinal pathology may be missed due to the poor view secondary to the cataract. Such patients will present to the vitreoretinal surgeon requiring PPV after their initial cataract surgery.

The purpose of this study was to compare intraoperative and postoperative outcomes of simultaneous versus sequential cataract surgery and PPV in terms of visual acuity, refractive outcomes, and intraoperative and postoperative complications.

## Methods

We conducted a retrospective chart review of electronic health records (EHR) of patients undergoing PPV and cataract surgery between March 2016 and May 2021 at the Wilmer Eye Institute, Johns Hopkins Hospital. This study was approved by the institutional review board (IRB) at The Johns Hopkins University and conducted in accordance with the tenets of the Declaration of Helsinki. Written informed consent was waived by the IRB for this study. HIPAA regulations were followed.

We divided our study population into three different cohorts: PPV/CE were patients undergoing PPV followed by cataract surgery; PPV + CE were patients undergoing simultaneous PPV and cataract surgery; and CE/PPV were patients undergoing cataract surgery followed by PPV. Visual acuity (VA), refractive error, slit lamp and fundus examination findings, and surgical complications were assessed at pre-specified time points relative to the date of cataract surgery in PPV/CE, date of simultaneous surgery in PPV + CE, and date of cataract surgery in CE/PPV.

The electronic health record (Epic, Verona, WI, USA) was used to review patient demographics, history of prior surgeries, and intraoperative and postoperative complications. EHR review also included uncorrected distance visual acuity (UDVA), corrected distance visual acuity (CDVA), spherical equivalent (SEQ), and intraocular pressure (IOP). Data recorded included the preoperative visit, as well as the 1 day (UDVA and IOP only), 1 week (UDVA and IOP only), 1-month (POM1), 3-month (POM3), 6-month (POM6), 1-year (POM12), and 2-year (POM24) postoperative visits. Snellen visual acuity was converted to logarithm of minimum angle of resolution (logMAR) for statistical analysis. Due to the duration of postoperative recovery, the 1-month and 3-month (POM 1 + 3), as well as the 6-month to 24-month (POM6-24) refractive data were combined, ensuring that only unique values were included.

Preoperative optical biometry was obtained by optical coherence interferometry (IOLMaster 500 or IOLMaster 700, Carl Zeiss Meditec, Jena, Germany). The Holladay I + II, SRK/T, and Barrett formulas were used to calculate the power of the implanted intraocular lens (IOL) in the majority of cases.

Refractive prediction error (RPE) was calculated as the difference between actual postoperative SEQ and the predicted SEQ. Visual acuity (UDVA + CDVA) and refractive outcomes were analyzed starting from the 1 + 3 month visit and ending at the 2-year follow-up visit. Only patients who received a three-piece foldable acrylic IOL (MA50BM; Alcon Laboratories, Inc, Fort Worth, TX, USA), or a one-piece acrylic IOL (Acrysof SA60AT, Acrysof IQ SN60WF, or Acrysof Aspheric SA60WF; Alcon Laboratories, Inc, Fort Worth, TX, USA), were eligible for inclusion in visual and refractive calculations (373 eyes). Patients who had prior history of refractive surgery (34 eyes) or had no clearly defined target refraction (13 eyes) were excluded from the UDVA, SEQ, and RPE calculations. Patients with a postoperative refractive target for near vision (between -1.0 to -7.0 diopters) were excluded from the UDVA and SEQ analysis (100 eyes). Patients without a minimum of one postoperative follow-up visit that included manifest refraction measurements (83 eyes) were excluded from SEQ and RPE calculations. Patients with history of endophthalmitis or with other vision-limiting diseases (e.g., macula off retinal detachment, severe diabetic retinopathy with macular involvement) were excluded from visual and refractive analysis.

To assess whether including patients with emergent retinal pathologies such as rhegmatogenous retinal detachment (RRD) or tractional retinal detachment (TRD) had a considerable impact on our visual acuity and refractive data results, we performed a sub-group analysis to include only those with non-emergent indications for surgery such as epiretinal membrane (ERM) and vitreous opacities (VO). SEQ and RPE analyses were only done on this subgroup to avoid incorrect refractive data in patients with poorer visual acuity.

The primary outcomes examined included both visual (UDVA, CDVA) and refractive (SEQ, RPE) variables. Secondary outcomes included intraoperative and postoperative complications, as well as IOP measurements. Binary variables are presented as N (%), and continuous variables are presented as mean ± standard deviation. Differences in proportions of binary variables were assessed using the chi-squared test. For continuous data, normality was determined using the Shapiro Wilk test (*p* < 0.05 was categorized as non-normal data). For data with a normal distribution, analysis of variance (ANOVA) was used to determine significant differences in means among the three comparison groups. For non-normal data, the nonparametric Kruskal–Wallis test was used in substitution of the ANOVA test. A *p* value cutoff of < 0.05 was defined as statistically significant. Statistical analysis was performed using STATA version 16.1 (StataCorp, College Station, Texas, USA).

## Results

### Baseline demographics and indications for PPV surgery

Four hundred twenty-seven eyes of 404 patients were included in our study. There were 113, 261, and 53 eyes in the PPV/CE, PPV + CE, CE/PPV groups, respectively. Demographic and ocular characteristics of all three cohorts are shown in Table 1. There was a statistically significant difference in age in the PPV/CE, PPV + CE, CE/PPV groups ((59.9 ± 8.97, 64.9 ± 10.40, and 65.4 ± 9.33, respectively (*P* < 0.001)).

**Table 1:** Demographics and ocular characteristics of all three cohorts

Parameters	Sequential PPV followed by Cataract Surgery	Simultaneous PPV & Cataract Surgery	Sequential Cataract Surgery followed by PPV	*P* value
Number of eyes	113	261	53	
Number of patients	107	248	49	
Age, years				**< 0.001**
Mean (SD)	59.9 (± 8.97)	64.9 (± 10.40)	65.4 (± 9.33)	
Range	30–75	26–89	47–85	
Gender				0.580
Male	56 (52.3)	138 (55.6)	29 (59.1)	
Female	51 (47.7)	110 (44.4)	20 (40.9)	
Laterality				0.677
Right	57 (50.4)	136 (52.1)	30 (56.6)	
Left	56 (49.6)	125 (47.9)	23 (43.4)	
Diagnosis				**< 0.001**
ERM	25 (22.1)	123 (47.1)	13 (24.5)	
Macular hole	19 (16.8)	46 (17.6)	5 (9.4)	
Vitreous Opacities	7 (6.2)	23 (8.8)	8 (15.1)	
Rhegmatogenous RD	38 (33.6)	8 (3.1)	14 (26.4)	
Tractional RD	4 (3.5)	16 (6.1)	4 (7.5)	
Combined mechanism of RD^a^	2 (1.8)	11 (4.2)	2 (3.8)	
Silicone oil removal	2 (1.8)	16 (6.1)	0	
Vitreous hemorrhage	13 (1.8)	9 (3.4)	2 (3.8)	
Dislocated IOL/Lens fragments retrieval	1 (0.9)	8 (3.1)	3 (5.7)	
Endophthalmitis	1 (0.9)	0	2 (3.8)	
Traumatic cataract	1 (0.9)	1 (0.4)	0	

Table values are n (%), unless otherwise indicated

*PPV* Pars plana vitrectomy, *CE* Cataract extraction surgery, *ERM* Epiretinal membrane, *RD* Retinal detachment, *IOL* Intraocular lens, *SD* Standard deviation

^a^Rhegmatogenous and tractional mechanisms of retinal detachment

There was a statistically significant difference in the indication for PPV between the three groups (*P* < 0.001). The top indications for PPV in PPV/CE group were RRD (33.6%), ERM (22.1%), and macular hole (MH) [16.8%]. In PPV + CE group, ERM was the most frequent diagnosis (47.1%), followed by MH (17.6%), and VO (8.8%). In CE/PPV group, RRD was the most frequent diagnosis (26.4%), followed by ERM (24.5%) and VO (15.1%).

**Fig. 1:**
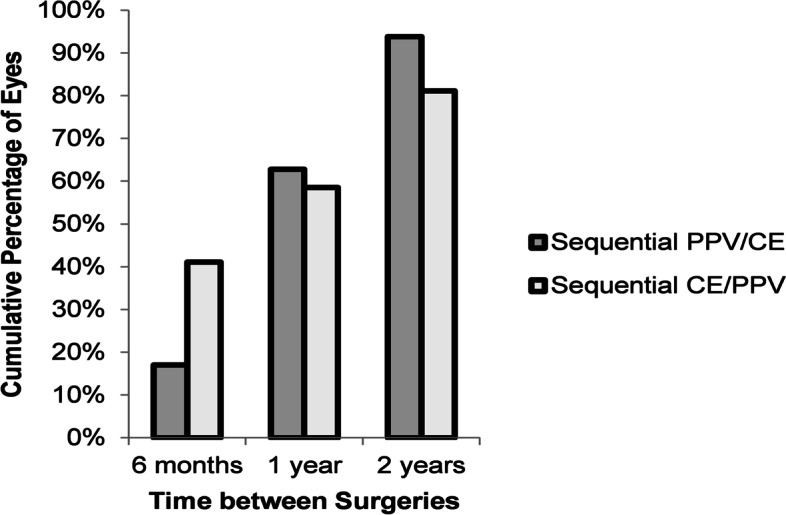
Cumulative percentage of eyes undergoing sequential surgery within the specified time frames. PPV/CE = pars plana vitrectomy followed by cataract extraction surgery; CE/PPV = cataract extraction surgery followed by pars plana vitrectomy

In the PPV/CE, the mean time elapsed between PPV and sequential CE was 347 ± 209 days (range 26 to 1108 days). 62.8 and 93.8% of patients in this group underwent cataract surgery within one year and two years following PPV, respectively (Fig. 1). Older age was associated with a shorter interval between PPV and subsequent CE. Patients who underwent CE within 6 months after PPV were older (63.5 ± 8.8 years) than those between 6 months and a year (mean age 60.3 ± 8.6 years), followed by those after one year ([mean age 57.8 ± 8.9 years]; *P* = 0.08).

In the CE/PPV, the mean interval between patients undergoing CE and sequential PPV surgery was 382 ± 387 days (range, 9 to 1491 days). 58.5 and 81.1% of patients in this group needed sequential PPV within 1 year and 2 years following cataract surgery, respectively (Fig. 1).

### Visual outcomes

Improvement in the UDVA of the whole patient cohort was seen at every follow-up visit compared to preoperative measures (Table 2). Preoperative UDVA values were comparable in the PPV/CE, PPV + CE, CE/PPV groups (logMAR 1.06 ± 0.64, 1.16 ± 0.64, and 1.03 ± 0.64, respectively (*P* = 0.67)). There was a statistically significant difference in the UDVA of the PPV/CE, PPV + CE, CE/PPV groups (logMAR 0.44 ± 0.45, 0.69 ± 0.60, and 0.60 ± 0.65, respectively) at the POM1 visit (*P* = 0.02). However, the difference was not statistically significant at POM3 (*P* = 0.35), POM6 (*P* = 0.50), or POM12 visit (*P* = 0.24).

**Table 2:** Comparison of visual acuity and intraocular pressure outcomes over time of all three cohorts

	**Sequential PPV followed by Cataract Surgery**		**Simultaneous PPV & Cataract Surgery**		**Sequential Cataract Surgery followed by PPV**		
**Parameters**	**n**	**Mean ± SD**	**n**	**Mean ± SD**	**n**	**Mean ± SD**	***P* value**
UCVA (logMAR)							
Preop	18	1.06 ± 0.64	62	1.16 ± 0.64	16	1.03 ± 0.70	0.67
POM 1	33	0.44 ± 0.45	137	0.69 ± 0.60	19	0.60 ± 0.65	**0.02**
POM 3	14	0.65 ± 0.62	92	0.77 ± 0.73	10	0.43 ± 0.43	0.35
POM 6	14	0.39 ± 0.34	62	0.64 ± 0.70	8	0.80 ± 0.79	0.50
POM 12	12	0.25 ± 0.16	48	0.68 ± 0.81	4	0.50 ± 0.37	0.24
POM 24	10	0.34 ± 0.32	24	0.37 ± 0.41	N/A		0.85
CDVA (logMAR)							
Preop	84	0.62 ± 0.50	238	0.73 ± 0.64	38	0.75 ± 0.66	0.80
POM 1	65	0.34 ± 0.40	190	0.65 ± 0.61	31	0.55 ± 0.60	**< 0.001**
POM 3	43	0.41 ± 0.47	152	0.64 ± 0.68	20	0.49 ± 0.62	0.11
POM 6	49	0.39 ± 0.45	116	0.47 ± 0.50	22	0.53 ± 0.70	0.38
POM 12	39	0.25 ± 0.34	118	0.53 ± 0.68	17	0.44 ± 0.48	**0.04**
POM 24	33	0.21 ± 0.47	74	0.50 ± 0.70	10	0.54 ± 0.67	0.07
IOP (mmHg)							
Preop	109	15.91 ± 4.22	256	15.25 ± 4.30	48	15.15 ± 3.80	0.93
POM 1	90	14.30 ± 3.80	234	14.85 ± 5.25	44	13.16 ± 3.31	0.16
POM 3	57	13.72 ± 3.26	177	14.66 ± 4.53	34	14.24 ± 3.57	0.46
POM 6	58	14.86 ± 5.33	177	14.83 ± 4.42	34	14.79 ± 3.41	0.94
POM 12	55	15.10 ± 4.76	143	15.24 ± 5.24	34	14.88 ± 5.89	0.64
POM 24	44	15.11 ± 3.53	92	16.15 ± 6.12	25	15.52 ± 3.72	0.68

*PPV* Pars plana vitrectomy, *UCVA* Uncorrected visual acuity, *CDVA* Corrected distance visual acuity, *IOP* Intraocular pressure, *SD* Standard deviation, *logMAR* Logarithm of the minimum angle of resolution, *POM* Postoperative month, *N/A* Not available

Improvement in the CDVA of the whole patient cohort was seen at every follow-up visit compared to preoperative measures. Preoperative CDVA values were comparable in the PPV/CE, PPV + CE, CE/PPV groups (logMAR 0.62 ± 0.50, 0.73 ± 0.64, and 0.75 ± 0.66, respectively (*P* = 0.80)). There was a statistically significant difference in CDVA of the PPV/CE, PPV + CE, CE/PPV groups (logMAR 0.34 ± 0.40, 0.65 ± 0.61, and 0.55 ± 0.60, respectively) at POM1 (*P* < 0.001), and at the POM12 visits (logMAR 0.25 ± 0.34, 0.53 ± 0.68, and 0.44 ± 0.48; *P* = 0.04).

There was no statistically significant difference in postoperative UDVA and CDVA of the PPV/CE, PPV + CE, CE/PPV groups who had a diagnosis of ERM or VO at any postoperative visit (Table 3).

**Table 3:** Comparison of visual acuity and refractive outcomes of eyes with epiretinal membrane and vitreous opacities

	**Sequential PPV followed by Cataract Surgery**		**Simultaneous PPV & Cataract Surgery**		**Sequential Cataract Surgery followed by PPV**		
**Number of eyes**^**a**^	**30**		**146**		**21**		
**Parameters**	**n**	**Mean ± SD**	**n**	**Mean ± SD**	**n**	**Mean ± SD**	***P* value**
UCVA (logMAR)							
Preop	5	0.81 ± 0.23	27	0.72 ± 0.42	6	0.45 ± 0.33	0.11
POM 1	7	0.23 ± 0.44	72	0.44 ± 0.31	7	0.35 ± 0.30	0.15
POM 3	2	0.39 ± 0.12	42	0.49 ± 0.50	3	0.37 ± 0.55	0.73
POM 6	5	0.28 ± 0.06	34	0.36 ± 0.30	4	0.45 ± 0.37	0.99
POM 12	5	0.24 ± 0.14	21	0.42 ± 0.43	1	0.3	0.80
POM 24	4	0.20 ± 0.14	10	0.20 ± 0.15	N/A		0.99
CDVA (logMAR)							
Preop	24	0.38 ± 0.24	12	0.41 ± 0.34	16	0.42 ± 0.34	0.77
POM 1	19	0.24 ± 0.31	98	0.38 ± 0.31	11	0.35 ± 0.29	0.14
POM 3	11	0.27 ± 0.27	76	0.37 ± 0.44	5	0.34 ± 0.37	0.48
POM 6	16	0.19 ± 0.18	64	0.29 ± 0.33	11	0.22 ± 0.26	0.65
POM 12	12	0.11 ± 0.20	64	0.30 ± 0.39	6	0.29 ± 0.36	0.06
POM 24	11	0.17 ± 0.19	36	0.25 ± 0.38	6	0.33 ± 0.29	0.57
SEQ (D)							
Preop	10	-5.6 ± 5.79	70	-0.41 ± 2.65	N/A		**0.002**
POM 1 + 3	9	0.03 ± 0.74 (Median 0.13)	44	-0.24 ± 0.58 (Median -0.25)			0.09
POM 6–24	6	0.21 ± 0.81 (Median 0.13)	37	-0.26 ± 0.71 (Median -0.38)	N/A		0.160
RPE (D)							
POM 1 + 3	17	0.05 ± 0.86 (Median 0.18)	51	-0.03 ± 0.66 (Median 0.05)	N/A		0.13
POM 6–24	10	0.25 ± 0.69 (Median 0.02)	44	-0.12 ± 0.95 (Median -0.10)	N/A		0.260

*UCVA* Uncorrected visual acuity, *CDVA* Corrected distance visual acuity, *SEQ* Spherical equivalent, *RPE* Refractive prediction error, *SD* Standard deviation, *D* Diopters, *logMAR* logarithm of the minimum angle of resolution, *POM* Postoperative month, *N/A* Not available

^a^Number of eyes before application of exclusion criteria

### Refractive outcomes

Due to the retrospective nature of our study, and the majority of follow-up visits guided more towards retinal examinations as the PPV was the latter of the two surgeries, limited numbers of eyes in CE/PPV had refractive data and were not included in the refractive data calculations. Only data for patients with ERM or VO were included in the SEQ or RPE analysis.

SEQ and RPE data is presented in Table 3. There was no statistically significant difference in SEQ in the PPV/CE group (mean 0.03 ± 0.74 D) compared to the PPV + CE group (mean -0.24 ± 0.58D) at the combined POM1 + 3 visits (*P* = 0.09). There was no statistically significant difference in RPE in the PPV/CE group (mean 0.05 ± 0.86 D) compared to the PPV + CE group (mean -0.03 ± 0.66D) at the combined POM1 + 3 visits (*P* = 0.13). This trend continued for the duration of follow-up visits (at POM6-24).

**Fig. 2:**
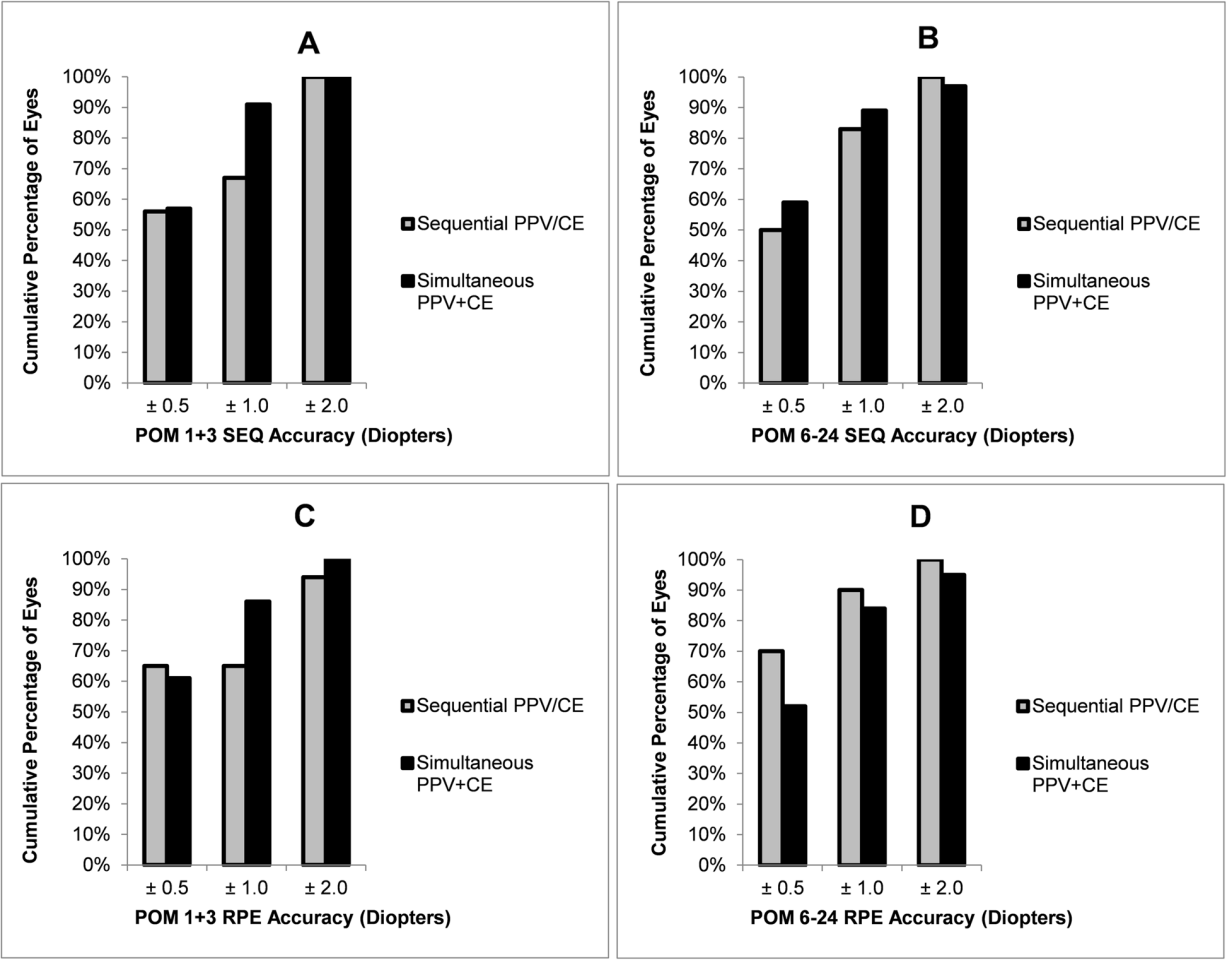
Comparison of postoperative month (POM) 1 + 3 (**A**), POM6-24 (**B**) spherical equivalent (SEQ) accuracy and POM1 + 3 (**C**), POM6-24 (**D**) refractive prediction error (RPE) accuracy within ± 0.5 D, 1.0 D, and 2.0 D in eyes with epiretinal membrane and vitreous opacity diagnoses. PPV/CE = pars plana vitrectomy followed by cataract extraction surgery; PPV + CE = simultaneous cataract extraction surgery and pars plana vitrectomy; POM = postoperative month

At the POM1 + 3 visits, SEQ was within ± 1.0 D in 6 eyes (67%) in the PPV/CE group, and 40 eyes (91%) in the PPV + CE group. SEQ was within ± 2D in 9 eyes (100%) in the PPV/CE group, and 44 eyes (100%) in the PPV + CE group (Fig. 2).

At the POM1 + 3 visits, RPE was within ± 1.0 D in 11 eyes (65%) in the PPV/CE group, and 44 eyes (86%) in the PPV + CE group. RPE was within ± 2D in 16 eyes (94%) in the PPV/CE group, and 51 eyes (100%) in the PPV + CE group.

### Secondary outcomes

IOP measurements showed no statistically significant differences between our three cohorts across all follow-up visits (Table 2).

Intraoperative and postoperative complications are presented in Table 4. The most common postoperative complication in all 3 groups was posterior capsule opacification (PCO), seen in 30.9, 21.4, and 11.3% in the PPV/CE, PPV + CE, CE/PPV groups, respectively. ERM was seen in 8.8, 2.3, and 1.9% of eyes in the PPV/CE, PPV + CE, CE/PPV groups, respectively. Cystoid macular edema developed in 4.4% of eyes in the PPV/CE group versus 3.8% in the PPV + CE group.

**Table 4:** Comparison of intraoperative and postoperative complications of all three cohorts

Parameters	Sequential PPV followed by Cataract Surgery	Simultaneous PPV & Cataract Surgery	Sequential Cataract Surgery followed by PPV
Intraoperative Complications			
Posterior capsule tear	0	1 (0.4)	3 (5.7)
Lens fragments dropping into vitreous	0	2 (0.8)	0
IOL prolapse into anterior chamber	0	1 (0.4)	0
Postoperative Complications			
Cornea			
Corneal edema	1 (0.9)	0	0
Anterior chamber			
Silicone oil in chamber	1 (0.9)	2 (0.8)	0
Hyphema	0	0	1 (1.9)
Iris			
Synechiae	0	2 (0.8)	0
Iris bombe	0	1 (0.4)	0
Vitreous			
Vitreous hemorrhage	1 (0.9)	3 (1.1)	0
Posterior vitreous detachment	1 (0.9)	0	0
Vitreous syneresis	0	0	0
Retina			
ERM	10 (8.8)	6 (2.3)	1 (1.9)
CME	5 (4.4)	10 (3.8)	0
Repeat PPV			
One repeat PPV	12 (10.6)	16 (6.1)	6 (11.3)
More than one repeat PPV	4 (3.5)	1 (0.4)	3 (5.0)
Posterior capsule opacification	35 (30.9)	56 (21.4)	6 (11.3)

Table values are n (%)

*PPV* Pars plana vitrectomy, *IOL* Intraocular lens, *ERM* Epiretinal membrane, *CME* Cystoid macular edema

## Discussion

This study compared the visual acuity and refractive outcomes, along with intraoperative and postoperative complication rates between PPV/CE, PPV + CE, and CE/PPV. In our patient population, VA outcomes and postoperative complications incidence did not vary across all three groups. An improvement in VA measurements was seen in all three groups when compared to baseline. Similar satisfactory refractive outcomes was seen in the ERM and VO sub-group.

The risk of developing lens opacification or progression of cataracts is recognized as one of the most frequent postoperative complications following PPV. Approximately, 68 to 100% of patients with vitrectomized eyes develop lens opacification or progression of their cataracts at their 2-year follow-up [1, 2, 9, 10]. This may be due to an increase in oxygen tension and subsequent oxidative damage to the lens following vitreous removal, accidental lens touch with intraocular instruments, and light toxicity induced by the operating microscope [3, 11, 12]. In our study, the timing of the sequential PPV/CE surgery highlighted the rapid progression of cataracts that is seen following PPV, with 62.8 and 93.8% of eyes having to undergo cataract surgery within 1 and 2 years following PPV, respectively (Fig. 1).

Patients who underwent CE within 6 months after PPV were older (63.5 ± 8.8 years) than those between 6 months and one year (mean age 60.3 ± 8.6 years), followed by those after one year ([mean age 57.8 ± 8.9 years]; *P* = 0.08). These findings were consistent with other reports that highlighted the impact of age on cataract progression following PPV, noting a 6- to 9- fold increase in cataract progression in patients older than 50 years in comparison to patients less than 50 years of age [10, 13]. Our findings may argue in favor of combined CEIOL and PPV in routine non-urgent cases, especially for patients older than 60 years of age.

In all three cohorts, improvement in UDVA and CDVA logMAR scores was observed in every follow-up visit when compared to baseline values. Although eyes in the PPV/CE group had better CDVA values, this only reached statistical significance in the POM1 and POM12 visits (*P* ≤ 0.001, *P* = 0.04, respectively).

We carried out a subgroup analysis to only include those with ERM or VO. Analysis of this subgroup did not show any significant difference in the UDVA or CDVA among the three groups. Based on the subgroup analysis, we believe that the sequence of the surgeries does not have a real impact on the postoperative UDVA or CDVA.

The SEQ and RPE outcomes were acceptable, with no significant difference seen in the PPV/CE and PPV + CE groups. Achieving a desirable refractive outcome is a challenge in patients undergoing PPV and cataract surgery, regardless of surgical timing. Some factors that must be taken into consideration when calculating postoperative refractive outcomes include anterior chamber depth (ACD), effective lens position (ELP), axial length (AL), use of intraocular gas tamponade, and type of formula used to determine IOL power. Obtaining an accurate AL measurement is regarded as the most influential step in calculating IOL power, with Olsen et al. [14] showing 54% of total prediction errors being caused by inaccuracies in measuring the AL. Obtaining an accurate AL measurement is more difficult and nuanced in patients with retinal pathology (such as RRD or significant ERM with macular edema) compared to the average population of cataract patients.

In our study, PCO was found to be the most frequent postoperative complication in all three cohorts. The frequency of PCO in PPV/CE was the highest, but was comparable in the PPV + CE and CE/PPV groups. This may be due to the fact that, in the PPV/CE group, there were higher incidents of posterior capsular fibrosis due to the previous PPV. Cataract surgeons may be more conservative in this patient population regarding thorough cleanup of the capsule at the end of the cataract surgery. This, in turn, may lead to higher rates of PCO. PCO has been identified as a common postoperative complication in 19.4 to 24.5% of eyes undergoing sequential PPV and cataract surgery [15, 16], and 21.5 to 23.5% of eyes following simultaneous phacovitrectomy [17, 18]. Various etiologies have been suggested for PCO development following simultaneous phacovitrectomy, including prolonged duration of surgery, extensive manipulation intraoperatively, and development of anterior chamber inflammatory reaction [19]. In a longitudinal cohort study investigating the development of PCO in patients undergoing phacovitrectomy with and without posterior capsulotomy, Shin et al. [20] noted a 6.4-fold increase in PCO incidence in the non-capsulotomy group in comparison to the capsulotomy group. Performing a posterior capsulotomy at the time of phacovitrectomy surgery may offer a better fundus view for the vitreoretinal surgery and reduce postoperative visits for management of PCO.

Strengths of this study include the large number of patient records reviewed in considerable detail, analysis of all three different possible combinations of PPV and cataract surgery, and two year follow-up duration. Limitations of this study include the retrospective study design. Also, determining axial length in the presence of retinal detachment is challenging and can be unreliable. Additionally, using spherical equivalent data did not allow us to determine the potential increased astigmatism due to the surgical procedure. Lastly, the procedures were performed by different surgeons with likely variations in surgical technique.

In conclusion, simultaneous PPV and cataract surgery demonstrated similar improvements in visual acuity and refractive outcomes. Simultaneous and sequential PPV/cataract surgery showed comparable intraoperative and postoperative complication profiles. As advanced patient age played a significant factor in the timing of sequential surgery and hastened cataract development requiring extraction following PPV, a simultaneous approach may be a suitable option for patients over the age of 60. Such an approach may avoid the likely deterioration in vision that arises with the progression of cataracts following PPV while eliminating the need for a second surgery.

## Data Availability

The datasets generated and/or analyzed during the present study are available from the corresponding author upon reasonable request.
